# Tert-butyl-(4-hydroxy-3-((3-(2-methylpiperidin-yl)propyl)carbamoyl)phenyl)carbamate Has Moderated Protective Activity in Astrocytes Stimulated with Amyloid Beta 1-42 and in a Scopolamine Model

**DOI:** 10.3390/molecules25215009

**Published:** 2020-10-29

**Authors:** Raúl Horacio Camarillo-López, Maricarmen Hernández Rodríguez, Mónica Adriana Torres-Ramos, Ivonne Maciel Arciniega-Martínez, Iohanan Daniel García-Marín, José Correa Basurto, Juan Vicente Méndez Méndez, Martha Cecilia Rosales-Hernández

**Affiliations:** 1 Laboratorio de Biofísica y biocatálisis, Escuela Superior de Medicina, Instituto Politécnico Nacional, Plan de San Luis y Diaz Mirón s/n, 11340 Ciudad de México, Mexico; rulojadfarij@gmail.com (R.H.C.-L.); dra.hernandez.ipn@gmail.com (M.H.R.); danielgarciaesm@gmail.com (I.D.G.-M.); 2 Unidad Periférica de Neurociencias, Facultad de Medicina UNAM-Instituto Nacional de Neurología y Neurocirugía, MVS-SSA, Insurgentes sur 3877, La Fama, Tlalpan, 14269 Ciudad de México, Mexico; monica.atorres@gmail.com; 3 Laboratorio de Inmunidad de Mucosas, Escuela Superior de Medicina, Instituto Politécnico Nacional, Plan de San Luis y Díaz Mirón s/n, 11340 Ciudad de México, Mexico; ivonne.arciniega.77@gmail.com; 4 Laboratorio de Diseño y Desarrollo de Nuevos Fármacos e Innovación Biotecnológica, Escuela Superior de Medicina, Instituto Politécnico Nacional, Plan de San Luis y Díaz Mirón, 11340 Ciudad de México, Mexico; corrjose@gmail.com; 5 Centro de Nanociencias y Micro y Nanotecnologías, Instituto Politécnico Nacional. Av. Luis Enrique Erro s/n, Nueva Industrial Vallejo, Gustavo A. Madero, 07738 Ciudad de México, Mexico; jmendezm@ipn.mx

**Keywords:** **M4** compound, amyloid beta peptide (Aβ_1-42_), β-secretase, Alzheimer’s disease, scopolamine, astrocyte cells

## Abstract

Alzheimer’s disease (AD) is a neurodegenerative disease with no cure nowadays; there is no treatment either to prevent or to stop its progression. In vitro studies suggested that tert-butyl-(4-hydroxy-3-((3-(2-methylpiperidin-yl)propyl)carbamoyl)phenyl) carbamate named the **M4** compound can act as both β-secretase and an acetylcholinesterase inhibitor, preventing the amyloid beta peptide (Aβ) aggregation and the formation of fibrils (fAβ) from Aβ_1-42_. This work first aimed to assess in in vitro studies to see whether the death of astrocyte cells promoted by Aβ_1-42_ could be prevented. Second, our work investigated the ability of the **M4** compound to inhibit amyloidogenesis using an in vivo model after scopolamine administration. The results showed that **M4** possesses a moderate protective effect in astrocytes against Aβ_1-42_ due to a reduction in the TNF-α and free radicals observed in cell cultures. In the in vivo studies, however, no significant effect of **M4** was observed in comparison with a galantamine model employed in rats, in which case this outcome was attributed to the bioavailability of **M4** in the brain of the rats.

## 1. Introduction

Alzheimer’s disease (AD) is a neurodegenerative disorder, which represents the principal cause of dementia. Although great efforts have been established to develop novel treatments, currently, the drugs employed to treat AD are still palliative.

Histopathological changes in the brains of AD patients are characterized by the presence of extracellular amyloid plaques composed principally of amyloid beta (Aβ) peptide, intracellular neurofibrillary tangles composed of phosphorylated Tau protein, and both neuronal and synaptic loss [1]. While the identity of the molecular species responsible for the initiation of AD pathology has been debated, genetic evidence indicates that aggregation of Aβ is a crucial step in the initiation of AD [2]. An imbalance between the production and the clearance of Aβ favors its increase and consequently, its accumulation. According to the amyloidogenic pathway, Aβ is produced from an amyloid β precursor protein by cleavage of the β-secretase enzyme. Once Aβ is released, it interacts with other monomers to produce oligomers (oAβ) and fibrils (fAβ), both species being highly neurotoxic. It was demonstrated that oAβ can produce neuronal cell death in hippocampal slice cultures at nanomolar concentrations [3]. Additionally, oAβ and fAβ can activate glial cells and establish a pro-inflammatory response [4,5,6].

Astrocytes, the most prevalent cell type in the brain along with the microglial cells, are key regulators of the brain inflammatory response. Reactive astrogliosis is a pathological characteristic found in the brains of AD patients. Interestingly, the degree of astrogliosis is correlated with cognitive decline [7,8]. Astrocytes can be stimulated by several forms; one of these is mediated by the recognition of Aβ_1-42_ by the Toll-like receptor 4 (TLR4) receptor [9,10]. It has been demonstrated that picomolar concentrations oAβ produces a TLR4 response that is time-dependent, leading to increased production of pro-inflammatory cytokines in astrocytic cultures. Furthermore, oAβ can induce neuronal cell death in co-cultures of astrocytes and neurons by an autocrine/paracrine mechanism due to TLR4 signaling [9]. Activation of TLR4 in astrocytes by Aβ_1-42_ aggregates leads to the production of an inflammatory response involving tumoral necrosis factor alpha (TNFα), interleukin-1 beta (IL-1β), interleucine-6 (IL-6), and the overexpression of the glial fibrillary acidic protein (GFAP) [11,12]. Interestingly, the aforementioned cytokines have been found in the brains of AD patients [4,5,6]. All the mediators produced by the glial activation cause an increase in oxidative stress and neuronal damage, especially in the brain rich in cholinergic neurons [13,14].

Many studies have focused on discovering new drugs to reduce the neuroinflammation and also to prevent neuronal death and improve the quality of life of patients [15]. In this regard, a great number of natural compounds have been evaluated as inhibitors of Aβ aggregation such as polyphenols (i.e., resveratrol, curcumin) or structurally similar to polyphenols such as tetracyclines [16]; a quinone-bearing polyamine compound such as memoquin has also emerged as a promising anti-AD candidate [17]. One of the main reasons most of the anti-AD compounds have failed in clinical trials is because a not significant effect in cognition decline has been observed [17,18]. These outcomes could be related to both the permeability of these compounds across the blood-brain barrier and their high molecular weight.

Due to the multifactorial pathogenesis of AD, the use of a multimodal therapeutic intervention addressing several molecular targets of AD-related pathological processes seems to be the most practical approach to modify the course of AD progression [19]. For this reason, our research group designed a novel multi-target compound tert-butyl-(4-hydroxy-3-((3-(2-methylpiperidin-yl)propyl)carbamoyl)phenyl) carbamate named **M4**. By employing in vitro studies, **M4** showed inhibition of both β-secretase 1 (IC_50_ = 15.4 nM) and acetylcholinesterase (K_i_ = 0.17 μM). Additionally, **M4** was able to inhibit Aβ aggregation (85% inhibition of aggregation at 100 μM) and showed poor antioxidant effect (IC_50_ => 400 μM) [20]. Therefore, the aims of this work were to assess by in vitro studies whether **M4** prevented astrocyte cell death induced by Aβ_1-42_ and investigate the ability of the **M4** compound to inhibit amyloidogenesis using a scopolamine AD-like model in vivo.

## 2. Results

### 2.1. Synthesis and Purification of the ***M4*** Compound

**Scheme 1:**
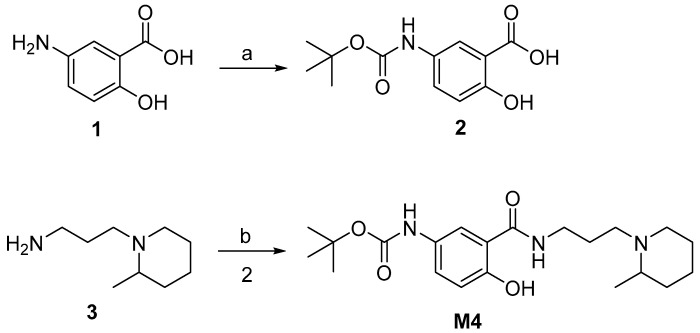
Synthesis of **M4**. Reagents and conditions; (a) di-tert-butyl dicarbonate (BOC_2_O), Ethyl acetate (EtOAc), triethylamine (TEA), room temperature (rt), 3 h; (b) 1-hydroxybenzotriazole hydrate (HoBt), *N*-(3-dimethylaminopropyl)-*N*-ethylcarbodiimide hydrochloride (EDCI), dry dichloromethane (DCM), rt, overnight.

In this work, we report the synthesis of tert-butyl-(4-hydroxy-3-((3-(2-methylpiperidin-yl)propyl)carbamoyl)phenyl)carbamate named **M4** by condensing *N*-(3-Aminopropyl)-2-pipecoline with 5-((tert-butoxycarbonyl)amino)-2-hydroxybenzoic acid, which is the BOC-protected form of 5-amino salicylic acid (Scheme 1). The condensation requires the activation of the carboxylic acid of compound 2 (5-amino-2-hydroxybenzoic acid) with a dehydrating agent such as 1-ethyl-3-(3-dimethylaminopropyl) carbodiimide (EDCI) in the presence of an additive like *N*-hydroxybenzotriazole (HoBt). The employment of additives during this type of condensation has been widely reported in the literature to increase reaction yields. It is reported that HoBt reacts with the *O*-acylurea to give the proper OBt activated ester, stabilizing the approach of the amine group via hydrogen bonding, and the enhancement in the reactivity of the activated ester is believed to lead to a higher yield of the amide product. Nevertheless, HoBt has also been proven to lead to the formation of by-products [21]. In our case, thin layer chromatography confirmed that starting materials led to a 100% conversion into the **M4** compound. During the liquid–liquid extraction performed with a KHCO_3_ solution, we realized that compound **M4** went into the aqueous phase to a moderate extent, which lowered the expected yield after column chromatography purification.

### 2.2. Identification of Fibrilar Aβ_1-42_ by Atomic Force Microscopy

**Figure 1:**
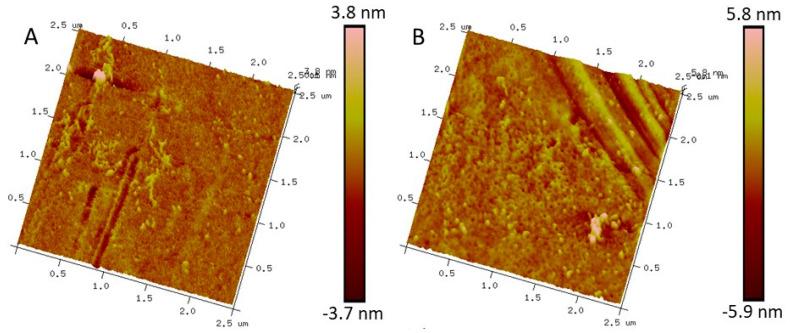
Atomic Force Microscopy image (2.5 × 2.5 µm^2^) of Aβ_1-42_ after 48 h of incubation were obtained using ScanAsyst mode (Bruker, USA). (**A**) Isolated fibers of Aβ_1-42_ can be seen. (**B**) A quasi-uniform layer of Aβ_1-42_ fibers can be observed.

It has been reported that during the stimulation of cells using Aβ_1-42_, its aggregation type is important [22]. Therefore, before stimulating the astrocytes with Aβ_1-42_, the formation and characterization of the fibril were done. Then, Aβ_1-42_ fibrils were identified by atomic force microscopy (scan area 2.5 × 2.5 µm^2^), obtained using the ScanAsyst mode, Bruker, Billerica, MA, USA). Figure 1 shows the images of Aβ_1-42_ incubated for 48 h at 37 °C in a quartz cell. In Figure 1A, it is possible to observe isolated fibers with an average diameter of 2.47 ± 0.86 nm formed by Aβ_1-42_. In Figure 1B, a quasi-uniform layer of fibers in the sample can be observed with the presence of some fibers above the base layer; these outcomes suggest that the Aβ_1-42_ peptide employed in astrocyte cell culture is in the fibrillary conformation, which seems to be in accordance with those reported in the literature [23,24].

### 2.3. Astrocyte Characterization

**Figure 2:**
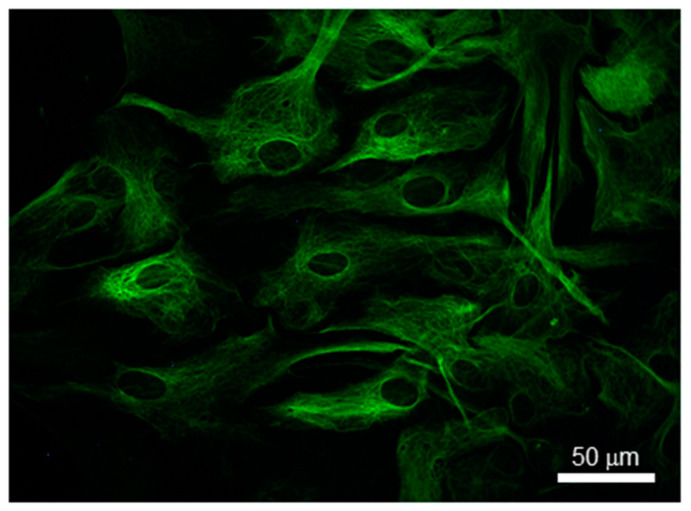
Astrocytes from a culture characterized by anti-GFAP labeling (green staining).

To evaluate the response of astrocytes by fibrillary Aβ_1-42_, the characterization of astrocytes obtained from primary rat cortical was undertaken. As can be seen in Figure 2, the astrocytes were characterized through immunostaining against the glial fibrillary acidic protein (GFAP). The astrocyte cultures were obtained with a 98% purity after the removal of microglial cells.

### 2.4. ***M4*** Compound Increases the Cell Viability of Astrocytes in the Presence of Aβ_1-42_

**Figure 3:**
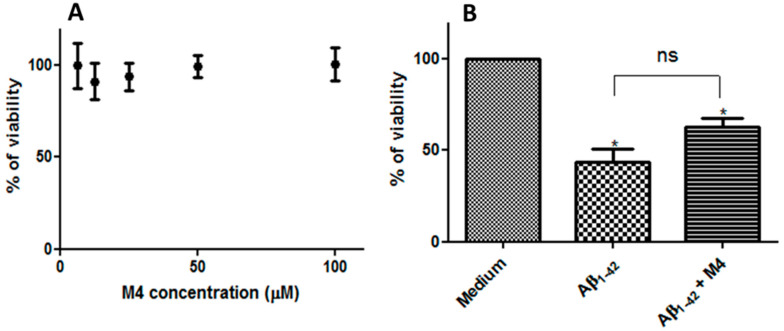
Viability of astrocyte cells from primary cultures among several treatments after 48 h of incubation, calculated by the 3-(4,5-Dimethylthiazol-2-yl)-2,5-Diphenyltetrazolium Bromide (MTT) assay. (**A**) Astrocyte viability in the presence of several concentrations of **M4**. The experiment was done in triplicate with *n* = 8. (**B**) Astrocyte activation with 30 μM Aβ_1-42_ peptide and the protective effect of the **M4** compound at 100 μM. The experiment was done in triplicate with *n* = 3. Statistical differences were calculated by one-way ANOVA, followed by Tukey’s test, and considering significant differences with * *p* < 0.05, (between medium and treatments).

As can be seen in Figure 3A, **M4** showed 100% of cell viability at 100 μM, suggesting no cytotoxicity related to this compound. When the astrocytes were treated first with the Aβ_1-42_ peptide, a significant decrease in cell viability was observed (43.78 ± 7.17%); however, when Aβ_1-42_ was added together with the **M4** compound, an improvement in the cell viability was observed (62.98 ± 4.92%), as a result of these outcomes, it could be possible that **M4** protected the astrocytes against the toxicity induced by Aβ_1-42_ (Figure 3B), albeit no significant difference was obtained between these groups.

### 2.5. ***M4*** Compound Protect against Reactive Oxigen Species (ROS) Produced by Aβ_1-42_ in Astrocyte Culture Cells

**Figure 4:**
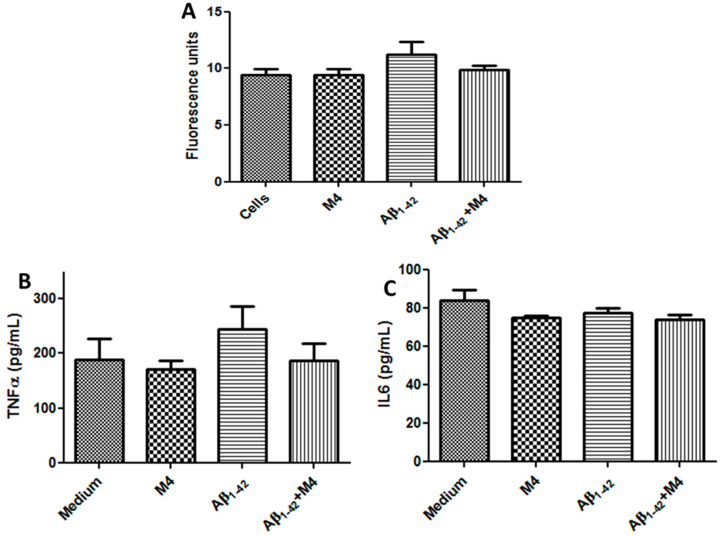
Changes in Reactive Oxigen Species (ROS) and pro-inflammatory cytokine levels in astrocytic primary cultures. (**A**) ROS determination by 2,7-Dichlorodihydrofluorescein Diacetate (DCFH-DA). The cells were treated with 100 μM of **M4** compound, 30 μM of Aβ_1-42_, and 30 μM of Aβ_1-42_ + 100 μM of **M4** compound for 48 h, and after treated with DCFH-DA (20 μM), the experiment was duplicated with *n* = 3. (**B**) TNF-α levels in the medium from astrocyte culture after 48 h of incubation with 100 μM of **M4** compound, 30 μM of Aβ_1-42_, and 30 μM of Aβ_1-42_ + 100 μM of **M4**. (**C**) IL-6 levels in the medium from astrocyte culture after 48 h of incubation with 100 μM of **M4** compound, 30 μM of Aβ_1-42_, and 30 μM of Aβ_1-42_ + 100 μM of **M4**. The results are presented as the mean ± SE. The experiment was duplicated with *n* = 2. Data were analyzed by one-way ANOVA followed by Tukey’s test and considering significant differences with *p* < 0.05.

As presented in Figure 4A, no difference in fluorescence was observed between control cells and those treated with **M4**, so this finding suggests that **M4** did not have any effect by itself on the astrocytes. In contrast, when the cells were treated with Aβ_1-42_, an increase in the fluorescence was observed with no significant difference. This change in fluorescence could be the result of an increased amount of ROS produced by the astrocytes, which in this case was promoted by the presence of the Aβ_1-42_ peptide. The assessment of **M4** along with Aβ_1-42_, showed a decrease in fluorescence compared to cells treated only with Aβ_1-42_, suggesting that the amount of ROS produced was reduced possibly due to the interaction between **M4** and the Aβ_1-42_ peptide.

### 2.6. Effect of ***M4*** Compound on IL-6 and TNF-α Astrocytes Activated with Aβ_1-42_

IL-6 and TNF-α levels were measured in the samples from culture cells of astrocytes treated with Aβ_1-42_ and **M4**. As shown in Figure 4B, the TNF-α levels increased in cells treated with Aβ_1-42_, however, no significant difference was observed in relation to cells in the medium. It was important to observe that treatment with the **M4** compound diminished the TNF-α levels in relation to cells treated with Aβ_1-42_, however, a significant difference was not obtained. As depicted in Figure 4C, IL-6 levels were not modified with any of the treatments employed. Therefore, it is believed that the moderated effect of **M4** in the in vitro studies could be related to the presence of Aβ_1-42_, and consequently employing **M4** as pretreatment of astrocyte cell cultures could possibly lead to a better outcome.

### 2.7. Moderated Activity of ***M4*** on Aβ_1-42_ Deposition

**Figure 5:**
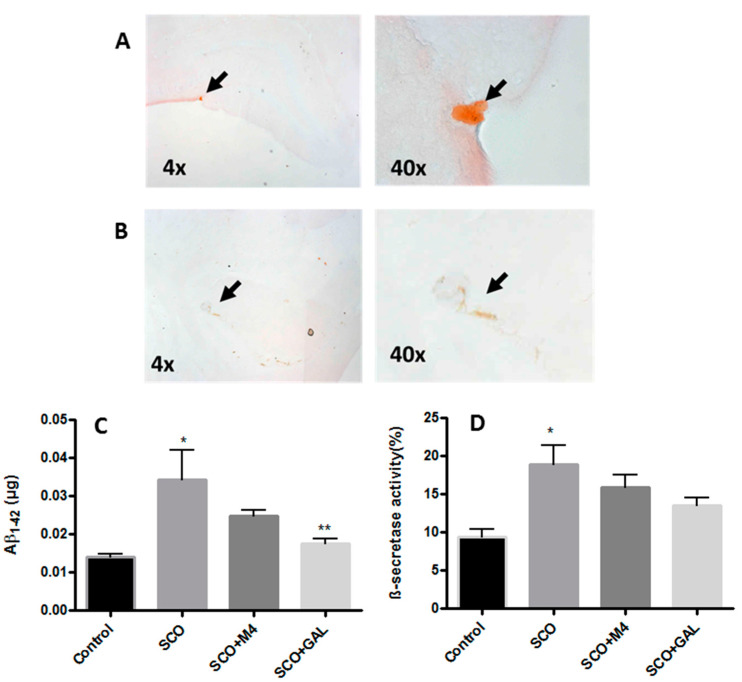
Effect of **M4** compound and galantamine administered intraperitoneally on Aβ production observed by Congo red stained in the coronal brain sections and Aβ_1-42_ and β-secretase activity quantification in the tissue of hippocampus. (**A**) Coronal section of brain samples obtained from rats treated with scopolamine plus **M4** compound. (**B**) Coronal section of brain samples obtained from rats treated with scopolamine and galantamine. (**C**) Aβ_1-42_ levels from the control samples, treated with scopolamine and scopolamine plus **M4** compound or galantamine (* *p* < 0.05 vs. control; ** *p* < 0.05 vs. sco). (**D**) β-secretase activity from control samples, treated with scopolamine and scopolamine plus **M4** compound or galantamine (* *p* < 0.05 vs. control). All experiment employed *n* = 8 and was done in triplicate.

The **M4** efficacy treatment was determined in the scopolamine-induced Aβ_1-42_ aggregation model. This model resembles the histopathological changes of AD and has been employed widely to evaluate the efficacy of novel compounds [25,26,27]. The Aβ plaques were evidenced by Congo red staining in all treatment groups, however, the **M4** and galantamine treatment groups showed the presence of these plaques; in the galantamine treatment, these plaques were shown to a lesser extent compared to **M4** (Figure 5A,B). Notwithstanding, both compounds showed the presence of Aβ plaques to a lesser extent compared with the scopolamine group, as reported in the literature [26].

Quantification of the Aβ_1-42_ levels are shown in Figure 5C, the Aβ_1-42_ levels increased in the scopolamine group relative to the control group with significant difference (* *p* < 0.05) [26], whereas in groups treated with **M4** and galantamine, the Aβ_1-42_ levels decreased, however, only the treatment with galantamine had a significant difference with the scopolamine group (** *p* < 0.05). These results were in accordance with those obtained for the β-secretase activity in which the scopolamine group increased the β-secretase activity relative to the control group (* *p* < 0.05). In groups treated with **M4** and galantamine, a decrease in β-secretase activity was observed without a significant difference (Figure 5D).

### 2.8. Moderated Activity of ***M4*** on Scopolamine-Induced Oxidative Stress

**Figure 6:**
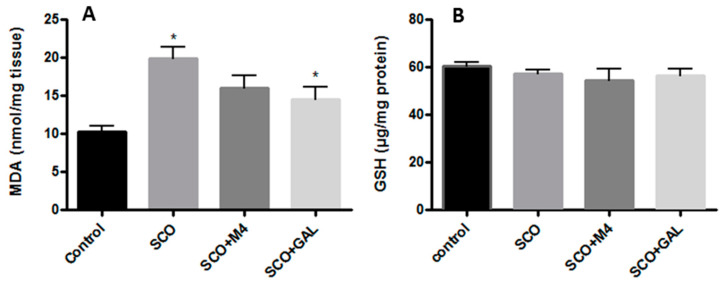
Oxidative stress effects of scopolamine and the **M4** compound administered intraperitoneally on malondialdehyde (MDA) and glutathione (GSH). (**A**) MDA levels from samples of the hippocampus tissue of rats without treatment (saline solution; control), treated with scopolamine and scopolamine with **M4** compound or scopolamine and galantamine, where a significant difference was obtained with respect to the control groups (* *p* < 0.05). (**B**) GSH quantification on samples with the same treatments as MDA, no significant difference was obtained between treatment groups. All experiments employed *n* = 8 and was done in triplicate.

To study the effect of the **M4** compound on scopolamine-induced oxidative stress, the malondialdehyde (MDA) and glutathione GSH levels were measured (Figure 6). The MDA levels increased in the scopolamine group, showing a significant difference with the control group (* *p* < 0.05; Figure 6A), which is in accordance with the findings reported in the literature [26]. A reduction in the MDA levels of brain homogenates (monitored by the Thiobarbituric Acid Reactive Substances (TBARS) assay) was observed in the samples treated with **M4** or galantamine compared to the scopolamine group. In this regard, a statistically significant decrease was observed when the samples were treated with galantamine in comparison with those treated with the **M4** compound.

The GSH content in brain samples was determined by the Ellman’s method. As can be seen in Figure 6B, GSH levels for groups treated with **M4** and galantamine were both alike with no statistically significant difference between them.

## 3. Discussion

The design and evaluation of new compounds for the treatment of AD are one of the most relevant areas in medicine as no cure for this is known as yet [28]. Several studies have proposed different pharmacophore molecules to inhibit the AChE, BACE1, and Aβ aggregation [16]. Among the chemical groups found in the proposed pharmacophores can also be found in amine functional groups and aromatics rings [29]. Taking into consideration these characteristics, the compound **M4** was designed and evaluated previously as a multitarget drug by in vitro studies [20]. In this work, we studied the ability of **M4** to stabilize the Aβ_1-42_ peptide and consequently avoid the activation of astrocyte cells through in vitro studies. This work also evaluated the changes in biological markers in a scopolamine AD-like model.

The **M4** compound was synthetized and characterized chemically with moderate yield (44%). With the aim to prevent unwanted products, the **M4** synthetic process was redesigned as depicted in Scheme 1. In the first place, the reactivity of the amine group of 5-ASA was blocked by introducing the BOC protecting group. In a second step, the carboxylic acid of 5-ASA was activated in situ by employing EDCI and HoBt as coupling reagents. This strategy resulted in a more convenient synthetic pathway as the by-products were significantly reduced to the minimal and the time processing was also reduced to 3 and 48 h, respectively.

The evaluation of **M4** was conducted by means of in vitro studies employing astrocyte cell cultures as this type of cell is the most prevalent cell type in the brain [7]. These types of cells are important in the central nervous system as they exert a key role in essential physiological processes; moreover, these types of cells are widely employed in the study of several brain pathologies [30,31].

The presence of **M4** in the astrocyte cell cultures treated with Aβ_1-42_, showed a reduction in the death of astrocytes only by approximately 20% compared to the cells treated with Aβ_1-42_ alone. Therefore, the affinity of Aβ_1-42_ for the astrocyte’s receptor could be higher than **M4**. Moreover, it is suggested that Aβ_1-42_ could be interacting with the astrocyte’s membrane, looking for hydrophobic environments [32]. This hypothesis was correlated with cytokine production as Aβ_1-42_ increases the TNF-α production, as reported in the literature [33]. However, **M4** decreases the TNF-α production, but not enough to be statistically different from those cells treated only with Aβ_1-42_. The IL-6 levels were not modified, which could be related to the Aβ_1-42_ species because different cytokine production has been reported depending on the type of cells and the Aβ_1-42_ conformation [22,34]. Furthermore, the ability of cells to produce IL-6 is different after activation; more IL-6 have been identified in microglial cells than astrocytes [35]. This could be the reason why no differences were observed with the treatments.

It has been demonstrated that the scopolamine-induced Aβ_1-42_ aggregation model showed histopathological changes like those found in the brain of patients with AD [25,26]. For this reason, we decided to evaluate if **M4** had any effect on the Aβ_1-42_ production and oxidative stress in vivo after scopolamine treatment over 12 weeks. Employing the Congo red staining, it was possible to observe the Aβ aggregates, which diminished substantially in the group treated with galantamine compared to the **M4** group. These findings were in accordance with the Aβ_1-42_ levels monitored in the brains of rats treated with **M4**, in which a reduction was observed. In contrast, the reduction of Aβ_1-42_ levels observed in the brains of rats treated with galantamine was statistically significant compared to the scopolamine treatment group. In addition, a reduction of β-secretase activity was observed in the scopolamine versus **M4** compound group.

Regarding oxidative stress, the outcomes suggest that **M4** was not able to significantly reduce the lipoperoxidation levels. This behavior seems to be in accordance with the lack of antioxidant activity observed in the **M4** compound [20]. However, galantamine demonstrated better reduction in lipoperoxidation levels, but without significant difference in relation to the scopolamine group. These findings seem to be in accordance with those reported in the literature in which the antioxidant activity of galantamine has been demonstrated [36]. Regarding GSH levels, no statistically significant changes were observed for samples treated with **M4** and galantamine against the control groups.

**Figure 7:**
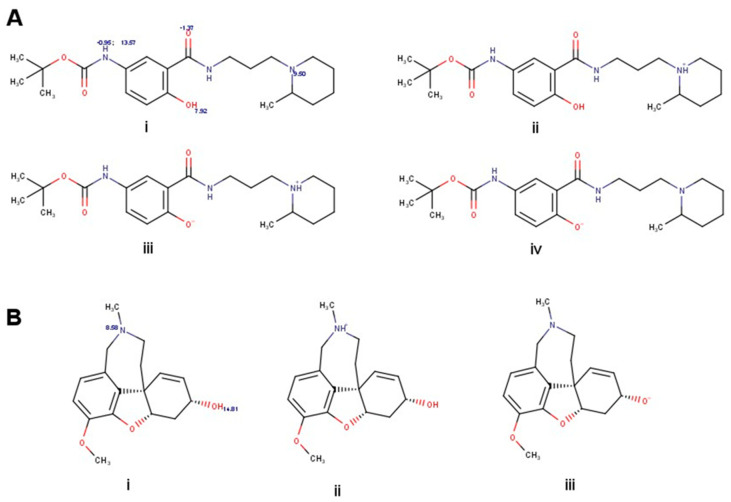
Main ionization states observed for the **M4** compound and galantamine across the whole range of the pH scale as determined by MarvinSchetch 20.13 software. (**A**) **M4** compound, pKa values (i), main chemical specie found below pH 7.6 (ii), main chemical specie found between pH 7.6–9.6 (iii), and main chemical specie found between pH 9.6–13.6 (iv). (**B**) Galantamine, pka values (i), main chemical specie found below pH 7.6 (ii), and main chemical specie found above pH 13.6 (iii).

The aforementioned outcomes are in agreement with those found in the literature, which stated that galantamine not only promotes the cholinergic neurotransmission in patients with AD, but also favors the non-amyloidogenic pathway for the PPA processing and additionally inhibits the β-secretase expression [37,38]. The poorly encouraging results obtained with **M4** could be explained by means of several factors, among which their characteristic short half-life and the low concentrations reaching the brain can be pointed out. In this regard, the protonation states of **M4** and galantamine were determined across the whole range of the pH scale with the aid of MarvinScketch 20.13 software. Despite both **M4** and galantamine compounds having a hydroxyl group and a tertiary amine, the **M4** exhibited more ionizable forms than galantamine; being eight ionized states for the former and only three for the latter. This could be explained by their pKa values and by the localization of these functional groups within the chemical structure of both compounds. In the case of the tertiary amine, the pKa value for **M4** was 9.5 while for galantamine it was 8.5. In the case of **M4**, the aromatic ring attached to the hydroxyl group provided a more stable state of the deprotonated form of the hydroxyl group leading to a pKa value of 7.92; in contrast, the deprotonated state of the hydroxyl group of galantamine cannot be easily stabilized, therefore, the pKa value reached as high as 14.91, which would explain why galantamine has only three protonated states compared to **M4**. Only the microspecies above 70% are presented in Figure 7.

Due to the ionization states, it might be possible that **M4** finds it even more difficult to cross the cell membranes when compared to galantamine. These results also help to explain why the **M4** showed better activity in vitro studies when Aβ_1-42_ was employed in its pure form [20] compared to the modest performance shown in cells and the in vivo model. To design better anti-Aβ compounds, the chemical characteristics of proposed molecules should be considered as this could be one of the reasons several designed compounds fail in clinical trials. Therefore, modification to chemical structure and increasing the dose employed are among the suggestions proposed to improve the performance of the **M4** compound.

## 4. Materials and Methods

### 4.1. Chemicals and General Characterization Methods

All chemical starting materials purchased from Sigma-Aldrich were used directly without further purification. All solvents were distilled prior to use. The purity of the synthesized compound was determined by ^1^H-NMR. ^1^H and ^13^C-NMR spectra were recorded on a Varian 300 MHz spectrometer (Varian Mercury-300 spectrometer, Varian, Palo Alto, CA, USA) in the deuterated solvents indicated; chemical shifts (δ) reported in ppm downfield of tetramethyl silane. Coupling constants (J) are reported in Hertz and rounded to 0.1 Hz. Splitting patterns are abbreviated as follows: singlet (s), doublet (d), triplet (t), quintet (quin), multiplet (m), broad (br), or a combination of these. Mass spectra were recorded by the staff at the Nanoscience and Micro-Nanotechnology Center at the Instituto Politecnico Nacional. High resolution mass spectra (HRMS) were recorded on a micOTOF-Q mass spectrometer (Bruker, Billerica, MA, USA). Thin layer chromatography (TLC) was performed using commercially available pre-coated plates (TLC Silica gel 60 F_254_, (Merck, San Francisco, CA, USA). Column chromatography was carried out using Merck Silica gel 60 (0.063–0.100 mm, (Merck, CA, USA).

#### Synthesis and Compound Characterization

Synthesis of **2** (5-(*N*-tert-Butoxycarbonylamino)-2-hydroxybenzoic Acid)

A solution of 5-aminosalicylic acid 1 (2.00 g, 13 mmol) in ethyl acetate (EtOAc) (100 mL) was placed in an ice bath, and degassed with N_2_ flow for 15 min. Thereafter, triethylamine (TEA) (2.8 mL, 20 mmol) was added dropwise followed by di-tert-butyl dicarbonate (4.4 mL, 19 mmol). The mixture reached room temperature and the reaction proceeded for 3 h. The solvent was removed under reduced pressure and the obtained residue was dissolved in distilled water. A white precipitate was obtained upon the addition of 5% HCl followed by vacuum assisted filtration. The precipitate was dried in an air-oven at 80 °C overnight to afford 5-((tert-butoxycarbonyl)amino)-2-hydroxybenzoic acid 2 as a white powder in an 89% yield; no further purification was required. Solubility: acetone, ethyl acetate, MeOH, DMSO, and THF. *R*_f_ 0.66 (MeOH). Decomposition was observed at 248 ± 1 °C. ^1^H-NMR (300 MHz, DMSO) δ 9.26 (s, 1H, N9-H amide), 7.98 (s, 1H, H-6), 7.47 (d, *J* = 8.7 Hz, 1H, H-4), 6.86 (d, *J* = 8.9 Hz, 1H, H-3), 1.45 (s, 10H, H-10).

Synthesis of **M4** (Tert-butyl-(4-hydroxy-3-((3-(2-methylpiperidin-yl)propyl)carbamoyl)phenyl)carbamate)

To a solution of 2 (2.00 g, 7.9 mmol) in dry DCM (100 mL), EDCI (2.56 g, 13.3 mmol) was added followed by 1-hydroxybenzotriazole hydrate (HOBt) (2.00 g, 13 mmol). This mixture was kept in an ice bath and degassed with N_2_ flow for 15 min. Thereafter, TEA (2.45 mL, 17.6 mmol) was added dropwise followed by *N*-(3-aminopropyl)-2-pipecoline **3** (2.3 mL, 13 mmol). The mixture reached room temperature and the reaction proceeded overnight protected from light under a N_2_ atmosphere. The organic mixture was washed with KHCO_3_ (5 × 100 mL) and dried with Na_2_SO_4_. After filtration, the solvent was removed under reduced pressure to afford a brown-like thick slurry. Solvent was perfectly removed from the crude mixture in a Schlenk line under vacuum overnight prior to silica gel column chromatography separation. The column was eluted with ethyl acetate, increasing the polarity up to ethyl acetate:methanol 2:1. Tert-butyl-(4-hydroxy-3-((3-(2-methylpiperidin-yl)propyl)carbamoyl)phenyl)carbamate, named as compound **M4**, was obtained from the column as a thick light-brown slurry, which was then subjected to solvent removal in a Schlenk line under vacuum overnight, to afford **M4** as a pale-yellow powder (44% yield). Solubility: acetone, CHCl_3_, ethyl acetate, MeOH, EtOH, DMSO, and H_2_O. Rf 0.1 (ethyl acetate: MetOH, 2:1). Decomposition was observed at 88–93 °C. ^1^H-NMR (300 MHz, CDCl_3_) δ 8.48 (s, 1H, NH-17), 7.68 (s, 1H, H-6), 7.17 (dd, *J* = 8.8, 2.4 Hz, 1H, H-4), 6.87 (d, *J* = 8.8 Hz, 1H, H-3), 6.48 (s, 1H, NH-7), 3.78–3.24 (m, 2H, H-8), 2.94 (m, 2H, H-10), 2.35 (m, 2H, H-11), 2.17–1.99 (m, 1H, H-15), 1.92–1.60 (m, 2H, H-9), 1.67 (d, *J* = 11.0 Hz, 2H, H-14), 1.55 (m, 2H, H-13), 1.48 (s, 9H, H-18), 1.36 (m, 1H, H-12), 1.07 (d, *J* = 6.3 Hz, 3H, H-16). ^13^C-NMR (300 MHz, CDCl3) δ 169.60 (C-7), 157.81 (C-17), 153.65 (C-2), 129.13 (C-5), 126.24 (C-4), 118.43 (C-3), 117.84 (C-6), 114.51 (C-1), 80.34 (C-18), 56.97 (C-15), 52.83 (C-10), 51.64 (C-11), 39.93 (C-8), 33.86 (C-14), 28.39 (C-19), 25.42 (C-9), 24.14 (C-13), 23.30 (C-12), 18.38 (C-16). IR (cm-1): NH (3295), C–H 2932, C=O (1705 amide I), C–CAr (1645), C=O (1550 amide II), C=O 1356, C–O–C 1240, C–OH (1159). HR-MS for [C_21_H_33_N_3_O_4_ + H]^+^, calculated 392.2471; found *m/z*: 392.2513 [M + H]^+^. All spectra of the characterization are presented in the Supplementary Material.

### 4.2. In Vitro Evaluation of Compound ***M4***

#### Formation and Determination of Fibrilar Aβ_1-42_

A total of 250 µg of human Aβ_1-42_ (Merck, Calbiochem^®^ cat: PP69-0.25 mg) diluted in 100 µL of MilliQ water was incubated in a quartz cell at 37 °C for 48 h. Two samples were taken from a previous solution and diluted (1:100), thereafter, the samples were placed on microscope slides and dried out at room temperature over 24 h in order to observe them using an atomic force microscope with a range of amplitude: 2–10 µm; Multi-touch mode.

### 4.3. Cortical Astrocyte Primary Culture

The primary cortical astrocyte culture was obtained according to the procedures previously reported [39]. All procedures complied with the Guidelines for the Care and Use of Laboratory Animals stipulated in the official norms for animal care in México (NOM-062-ZOO-1999 SAGARPA). The purity of the primary astrocyte culture was determined by double immunoflorescence with the anti-GFAP antibody (ZO334 Agilent, DAKO Laboratories, Santa Clara, CA, USA) for astrocytes in green-fluorescent dye (Alexa fluor 488) and anti-iba-1 antibody (ABCAM-AB178680) for microglia in red-fluorescent dye (Alexa fluor 594). Subsequently, the cells stained red of iba-1 were quantified for every 100 cells in a 10× field.

### 4.4. Cell Viability in the Presence of Compounds ***M4*** Was Determined by (3-(4,5-Dimethylthiazol-2-yl)-2,5-Diphenyltetrazolium Bromide) MTT Assay with and without Aβ_1-42_

The MTT assay correlates the reduction of diphenyltetrazolium bromide (yellow colored) into purple formazan with the viable cells present in the culture sample. For the MTT viability assay, 1 × 10^4^ cells per well were seeded in a 96-well plate. After 48 h incubation, the **M4** compound was added in an array of different concentrations (100, 50, 25, 12.5, and 6.25 μM) and incubated for another 48 h. The **M4** compound was dissolved in DMSO 0.01%. Thereafter, 20 μL of MTT (0.5mg/mL) was added to each well and incubated for 4 h in a CO_2_ incubator. Finally, the medium that contained the MTT solution was removed and replaced with 100 µL of DMSO at each well. The absorbance was then measured at 510 nm using a Multiskan SkyHigh Microplate Spectrophotometer (Thermo Fisher Scientific, Waltham, MA, USA).

The astrocyte activation was done with Aβ_1-42_, 1 × 10^4^ astrocytes cells were seeded in triplicate in a 96-well plate forming four groups with *n* = 8. After 48 h of cell stabilization, each group was supplemented with: (I) 100 µL of culture medium, (II) 100 µL of culture medium with DMSO 0.01%, (III) 100 µL of culture medium with Aβ_1-42_ (30 µM), (IV) 100 µL of culture medium with Aβ_1-42_ (30 µM) and **M4** compound at 100 µM, and (V) 100 µL of culture medium with the **M4** compound at 100 µM. Thereafter, the plate was incubated for 48 h, and finally the viability of cells was determined as described in the previous section by the MTT assay. The percentage of cell viability was calculated considering as 100% the viability of the cells with medium and DMSO 0.01%. All determinations were performed in triplicate with *n* = 8, the averages and standard errors (SE) were calculated. The results are presented as the mean ±SE.

### 4.5. Reactive Oxygen Species Quantification by 2,7-Dichlorodihydrofluorescein Diacetate (DCFH-DA) Assay

The intracellular ROS produced during the astrocyte’s activation were quantified by the oxidant-sensing probe 2,7-Dichlorodihydrofluorescein Diacetate (DCFH-DA), by adding 100 μL DCFH-DA at 20 μM to each well. Depending on the treatment, 1 × 10^4^ astrocytes were stimulated for 48 h with Aβ_1-42_ (100 µL of culture medium with Aβ_1-42_ at 30 µM). The treatment groups were similar as described in the previous section. After, the cells were incubated in an atmosphere with CO_2_ at 5% and 37 °C for 60 min. The presence of ROS promotes the oxidation of DCFH to 2,7-dichlorofluorescein DCF, which was measured by fluorescence at 485–528 nm excitation/emission, respectively, using a LS-45 spectrofluorometer (Perkin-Elmer, Waltham, MA, USA).

### 4.6. IL-6 and TNF-α Determination

Proinflammatory cytokine levels produced by the formerly stimulated cultures were quantified using the RayBio^®^ Human TNF-alpha enzyme-linked immunosorbent assay (ELISA) and RayBio^®^Human IL-6 ELISA kits (RayBioTech, Peachtree Corners, GA, USA). For both assays, 100 μL of the respective undiluted sample (medium from the treatments mentioned before I, II, III and IV), was added to 96-well plates previously precoated with the antibody and incubated for 2.5 h at 37 °C, according to manufacturer’s specifications. Detection antibodies were added and then horseradish peroxidase (HRP) conjugated streptavidin. After, the TMB substrate was added and incubated for 30 min, a stop solution was added, and the absorbance of the colored solution was measured as the endpoint at 450 nm using a MultiSkanSky microplate spectrophotometer (ThermoFisherScientific, Waltham, MA, USA). The reagents’ preparation and the respective washes were carried out following the provided user manual. The samples were measured in duplicate from two independent experiments and the TNFα/IL6 concentrations were calculated and plotted according to their respective standard curve provided in the kit using the Prism8 software (GraphPad Software Inc., San Diego, CA, USA).

### 4.7. Effect of ***M4*** Compound on the Scopolamine-Induced Aβ_1-42_ Aggregation Model

A total of 64 male Wistar rats of 250 +/−10 g weight, were used. The animals were purchased at the bioterium of the Institute of Neurobiology (INB) at the National Autonomous University of Mexico and kept at the Laboratory of Biophysics and Biocatalysis at the Superior School of Medicine. All animals remained in polysulfonate boxes for at least one week before the experiments. During the experiment, the animals were provided with light and dark cycles of 12 by 12 h, food and water (Rat Chow, Purina) ad libitum. A daily dose of saline solution was administrated to the control group, whereas scopolamine (2 mg/Kg) was administered intraperitoneally for a period of 12 weeks to three groups (named as; SCO, SCO + **M4** and SCO + GAL) of male Wistar rats. Six weeks after initiating the scopolamine administration, the rats also received the correspondent compound **M4** at 4 mg/Kg (SCO + **M4** group) while the galantamine group was administrated with 3 mg/Kg of galantamine (SCO + GAL group). Once the treatment for each group was concluded, the 16 animals in each group were sacrificed to perform the biochemical and histological studies described previously. The animal protocol was approved by the Research Committee for the Care and Use of Laboratory Animals (CICUAL) of the Escuela Superior de Medicina-IPN (Approval number: ESM.CICUAL-01/01-06-2015). All animal procedures were conducted considering the Mexican Official Standard (NOM-062-ZOO-1999) Technical Specifications for Production and Care and Use of Laboratory Animals. In this regard, the animals were first anesthetized with sodium pentobarbital (60 mg/kg) and then a transcardiac perfusion of 200 mL of 0.1 M PBS, pH 7.4 was applied. Eight out of 16 animals were employed for biochemical studies; therefore, the brains were extracted and divided into two hemispheres to facilitate the hippocampi dissection, which was then kept at −80 °C. From the remaining rats, one hemisphere was fixed in PBS containing 4% paraformaldehyde and was then cryoprotected in 50% sucrose to perform the histology studies. The hippocampus was dissected from the second hemisphere and was kept at −80 °C for quantification of βA_1-42_ by the ELISA assay.

### 4.8. Determination of Amyloid Deposits in Hippocampal Histological Sections

The brain tissue previously fixed in 4% paraformaldehyde for four days was washed with PBS and then cryoprotected with 50% saccharose for seven days. Thereafter, tissues were immersed in Tissue-Tek^®^ and were frozen with dry ice and were kept at −80 °C until further use. The hemisphere was cut into coronal sections of 40 µm thick using a Leica brand cryotome. The sections were placed in 24-well plates containing 0.1% sodium azide dissolved in PBS, and then were stored at 4 °C. A sample of each group was kept aside to perform Congo red staining. First, sodium azide was removed and the sections were incubated in a 20% Congo red solution in deionized water for 3 min at room temperature with stirring. Thereafter, samples were mounted on gelatinized slides and were dried for 24 h at 37 °C in an oven. Third, each of the slides was dehydrated by means of immersion in ethanol (ethanol 70° for 5 min, ethanol 96° for 5 min, ethanol 100° for 5 min), and subsequently rinsed with xylene for 5 min. Finally, each slide was placed in Entellan mounting medium (Merck Milipore, No. Cat. 107960).

### 4.9. β-Secretase Activity

The β-secretase activity present in brain homogenates was determined by means of the fluorescence resonance energy transfer (FRET) assay. The enzymatic kit employed (Sigma Aldrich, Toluca, México, Cat. No. CS0010) is based on the cleavage of PPA by the β-secretase, which leads to an increase in the fluorescence signal at λ emission = 405 nm and λ excitation = 320 nm, thereby allowing the determination of the activity of this enzyme in samples from brain homogenates. According to the kit specification, the enzyme was replaced by the brain homogenate used [40]. The percentage of hydrolysis was calculated according to the kit specification. The data are reported as the average of each experiment performed in triplicate with its standard error.

### 4.10. Quantification of Aβ_1-42_ by ELISA Assay

A sample (50 mg) of hippocampus tissue was placed in Tris-HCl buffer 10 mM containing 1% *v/v* Triton X-100, EDTA 1 mM and 0.1% *wt/v* protease inhibitor cocktail, then the mixture was homogenized by centrifugation at 500 rpm for 10 min at 4 °C. The appropriate amount of supernatant that contained 40 µg of protein (previously determined by Bradford assay) was diluted in 0.1 M pH 9.6 carbonate buffers up to a final volume of 50 µL. The mixture was incubated during 7 h at 4 °C in a plate with high protein binding wells (3590 COSTAR^®^). Afterward, three washes of two minutes each were carried out by employing PBS buffer 0.1 M pH 7.4 containing Tween 20 (PBST) at 0.05% *v/v*, subsequently, another three washes of two minutes each were performed using PBS buffer 0.1 M at a pH 7.4. To avoid unspecific binding sites, milk Svelty Figura 0%, Nestle^®^ at 6% *wt/v* in PBST was added and incubated for 2 h at 37 °C, finally, another washing sequence was performed. A total of 50 µL of anti-horseradish peroxidase (beta Amyloid Antibody MOAB-2NBP2-13075 NOVUS^®^) was added in a 1:500 dilution in PBST and the mixture was incubated during 2 h at 37 °C, thereafter, another sequence of washes was performed. Finally, the plate was developed with 50 µL of 2.7 mM O-phenilenediamine dihydrochloride (OPD) and the absorbance at 490 nm was quantified. A standard curve of absorbance at 490 versus human Aβ_1-42_ peptide (Calbiochem ^®^ cat: PP69-.25 mg) concentration (0.25, 0.125, 0.0625, 0.0312, 0.0156, 0.0078 µg) was employed for all calculations.

### 4.11. Malondialdehyde (MDA) Content Determination

The MDA levels were determined by the TBARS assay, for this purpose, 0.1 g of homogenized tissue in 1 mL of distilled water was centrifuged at 6000 rpm for 15 min. A total of 75 µL of the supernatant was diluted with 175 µL of 0.15 M Tris-HCl buffer at pH 7.4. After a 30 min period incubation at 37 °C, 500 µL of 3% (*w/v*) thiobarbituric acid (ATB) dissolved in 15% (*w/v*) trichloroacetic acid (ATC) was added to the mixture and the samples were incubated at boiling for 1 h. Finally, the samples were centrifuged at 6000 rpm for 15 min, the supernatant was removed and read at 540 nm. MDA content was expressed in nmol/mg tissue. The amount of protein was determined using the Bradford reagent (CAYMAN CHEMICAL) and the absorbances were read at 590 nm.

### 4.12. Quantification of Reduced Form of Glutathione (GSH)

The GSH content was determined according to procedures reported in the literature with some modifications [41]. A sample (20 mg) of hippocampus tissue was homogenized in 1 mL of 5% (*wt/vol*) metaphosphoric acid (MPA) in an ice bath employing a cell ultrasonic apparatus (VibraCell^®^) at 5000 rpm for 15 min. A sample from the supernatant (167 µL) was taken and diluted with 500 µL of phosphate buffer (0.1 M and pH 8), and the solution was supplemented with 10 µL of (5,5′-Dithio-bis)-2-nitrobezoic (DTNB) (1.99 mM). Quantification of the absorbance at a wavelength of 412 nm was performed with the aid of an ELISA reader (Multiskan-EX Thermo Scientific). A standard curve of Abs412 versus GSH concentration (1.25, 2.5, 5, 10, 20.40 µg of GSH; see Annex 13.1) was employed for all GSH calculations expressed in µg/mg of protein.

### 4.13. Statistical Analysis

The results are presented as the mean ± standard error SE. Data were analyzed by one-way ANOVA, followed by Tukey’s test and considering significant differences with *p* < 0.05, indicated as *; no statistically significant difference was indicated as ns. The analysis of the obtained data was performed with GraphPad Prism 7.0 software. Each experiment was done at least in duplicate.

## 5. Conclusions

The **M4** compound was designed as a multitarget molecule to be employed for the possible treatment of AD. However, the low effect observed in vitro, and in vivo studies might be related with the ionization state of **M4**, which in turn might be not only lowering the concentration of the compound that reaches the brain, but also reducing its half-life, despite being a small molecule compared to galantamine.

## Supplementary Materials

The following are available online. ^1^H-NMR, ^13^C-NMR, ^1^H-^1^H COSY 2D-NMR, ^1^H-^13^C HETCOR 2D-NMR, HRMS ESI^+^, HRMS-MS ESI^+^, and FTIR spectra of **M4**.
