# Supplementary material for: Tert-butyl-(4-hydroxy-3-((3-(2-methylpiperidin-yl)propyl)carbamoyl)phenyl)carbamate Has Moderated Protective Activity in Astrocytes Stimulated with Amyloid Beta 1-42 and in a Scopolamine Model
Source: Molecules. 2020 Oct 29;25(21):5009. doi: 10.3390/molecules25215009 (PMC7672627; doi:10.3390/molecules25215009)

Supplementary Material

Tert-butyl-(4-hydroxy-3-((3-(2-methylpiperidin-yl)propyl)carbamoyl)phenyl)carbamate Has Moderated Protective Activity in Astrocytes Stimulated with Amyloid Beta 1-42 and in a Scopolamine Model

Raúl Horacio Camarillo-López [1], Maricarmen Hernández Rodríguez [1], Mónica Adriana Torres-Ramos [2], Ivonne Maciel Arciniega-Martínez [3], Iohanan Daniel García-Marín [1], José Correa Basurto [4], Juan Vicente Méndez Méndez [5] and Martha Cecilia Rosales-Hernández [1,*]

[1] Laboratorio de Biofísica y biocatálisis, Escuela Superior de Medicina, Instituto Politécnico Nacional, Plan de San Luis y Diaz Mirón s/n, 11340 Ciudad de México, México; rulojadfarij@gmail.com (R.H.C.-L.); dra.hernandez.ipn@gmail.com (M.H.R.); danielgarciaesm@gmail.com (I.D.G.-M.)

[2] Unidad Periférica de Neurociencias, Facultad de Medicina UNAM-Instituto Nacional de Neurología y Neurocirugía, MVS-SSA. Insurgentes sur 3877, La Fama, Tlalpan, 14269 Ciudad de México, México; monica.atorres@gmail.com

[3] Laboratorio de Inmunidad de Mucosas, Escuela Superior de Medicina, Instituto Politécnico Nacional, Plan de San Luis y Díaz Mirón s/n, 11340 Ciudad de México, Mexico; ivonne.arciniega.77@gmail.com

[4] Laboratorio de Diseño y Desarrollo de Nuevos Fármacos e Innovación Biotecnológica, Escuela Superior de Medicina, Instituto Politécnico Nacional, Plan de San Luis y Díaz Mirón, 11340 Ciudad de México, México; corrjose@gmail.com

[5] Centro de Nanociencias y Micro y Nanotecnologías, Instituto Politécnico Nacional. Av. Luis Enrique Erro s/n, Nueva Industrial Vallejo, Gustavo A. Madero, 07738 Ciudad de México, México; jmendezm@ipn.mx

* Correspondence: marcrh2002@yahoo.com

* Correspondence: Martha Cecilia Rosales Hernández, marcrh2002@yahoo.com.

Figure S1

^{1}H-NMR spectrum of M4

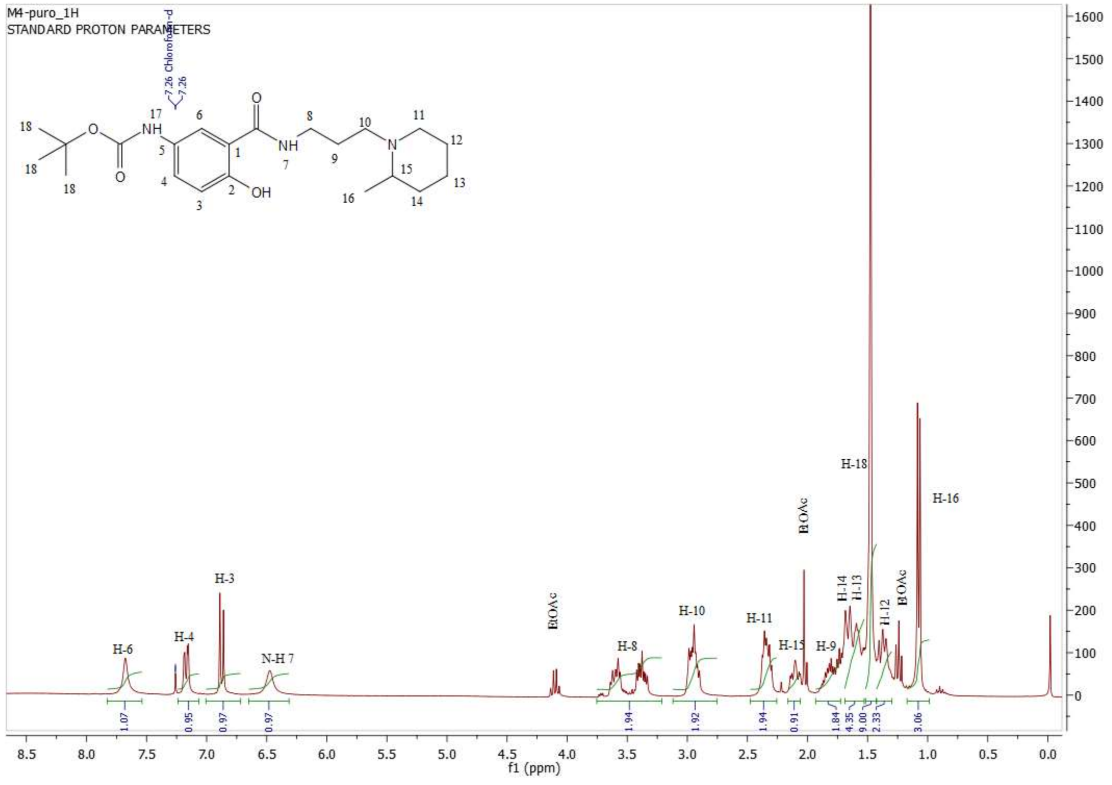

Figure S2

^{13}C-NMR spectrum of M4

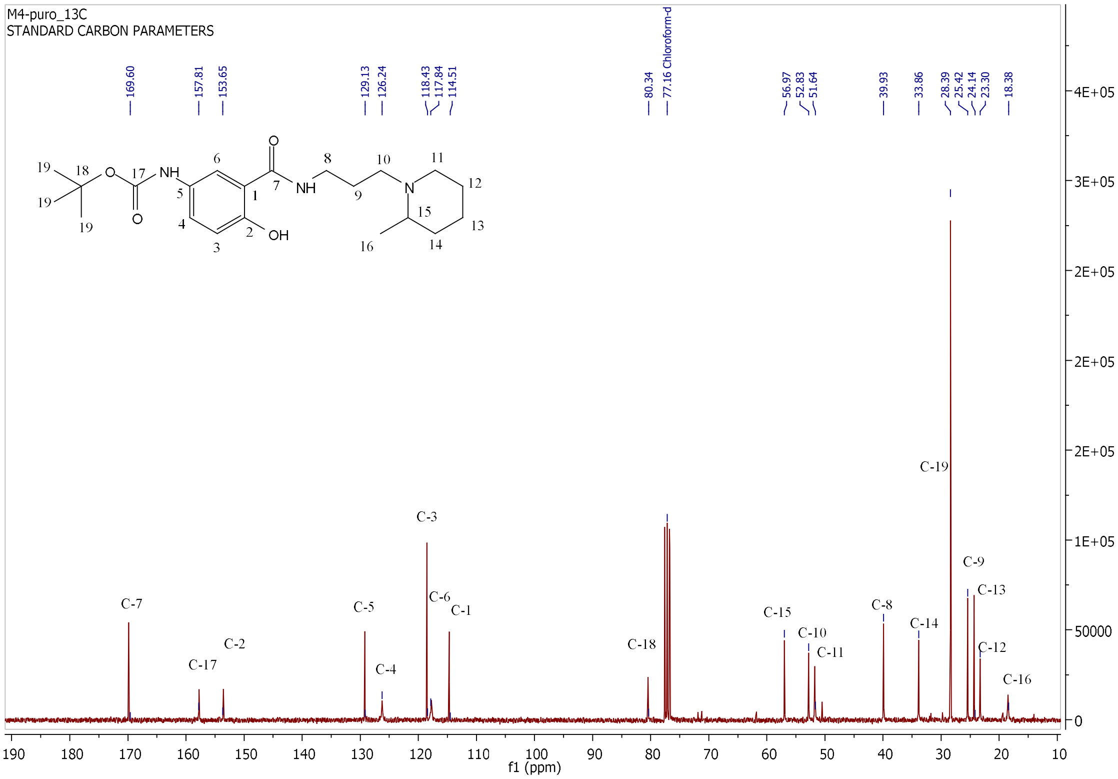

Figure S3

^{1}H-^{1}H COSY 2D-NMR spectrum of M4

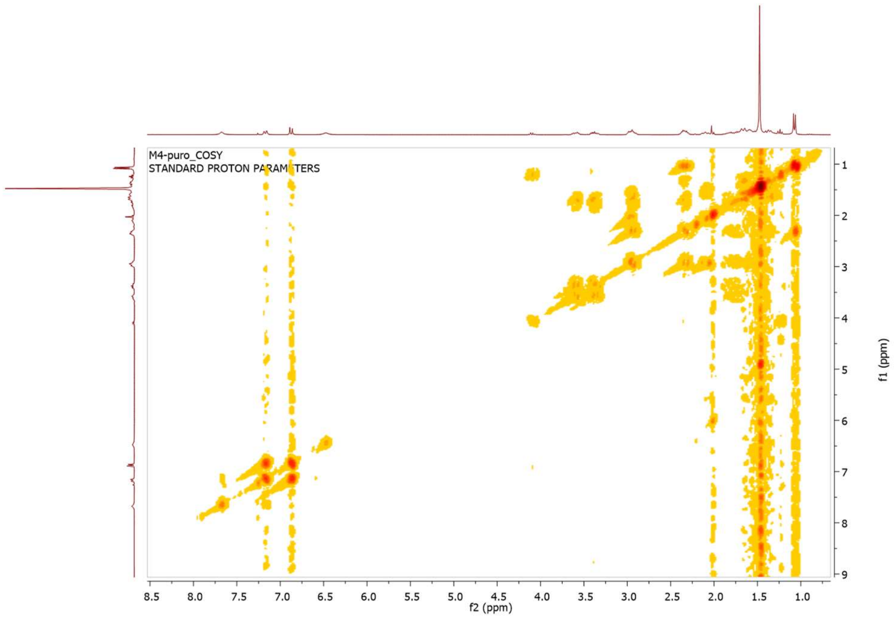

Figure S4

^{1}H-^{13}C HETCOR 2D-NMR spectrum of M4

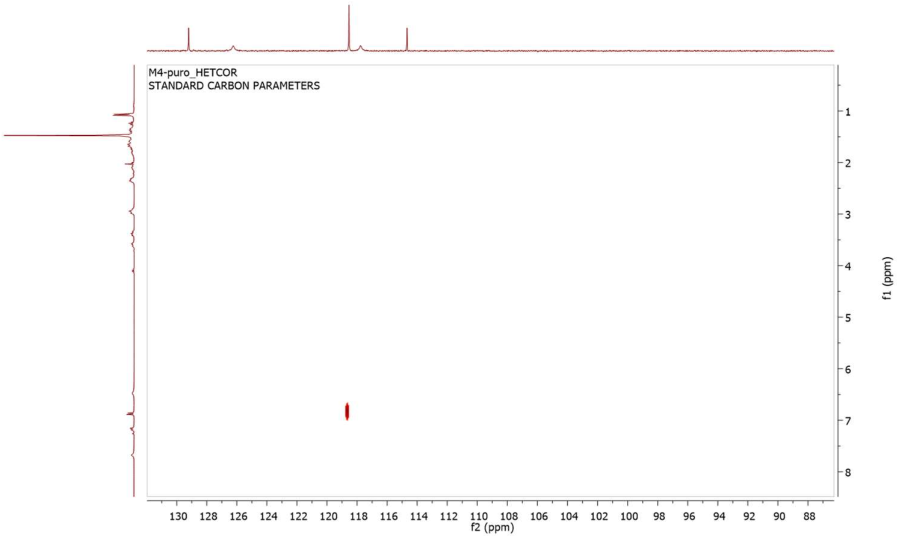

Figure S5

HRMS ESI$^+$ Spectrum of M4

Display Report

Analysis Info

Analysis Name	D:\Data\Jose Correa Basurto\112218_M-4__.d	Acquisition Date	11/22/2018 3:12:18 PM
Method	Tune low 25-sep-2018.m	Operator	Daniel Arrieta
Sample Name	112218_M-4__	Instrument	micrOTOF-Q 228888.10392
Comment			

Acquisition Parameter

Source Type	ESI	Ion Polarity	Positive	Set Nebulizer	0.4 Bar
Focus	Active	Set Capillary	4500 V	Set Dry Heater	180 °C
Scan Begin	50 m/z	Set End Plate Offset	-500 V	Set Dry Gas	4.0 l/min
Scan End	3000 m/z	Set Collision Cell RF	150.0 Vpp	Set Divert Valve	Source

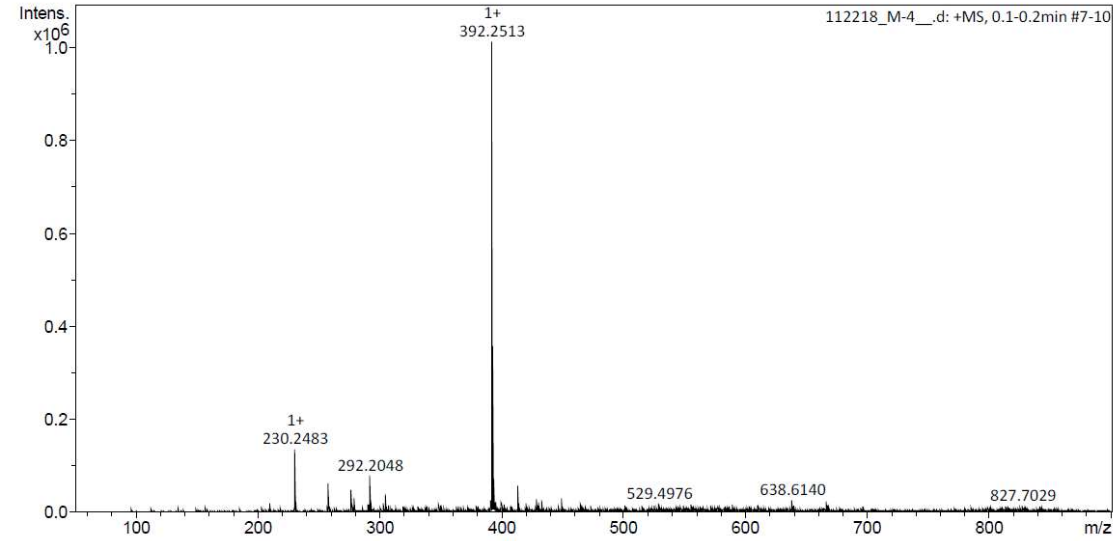

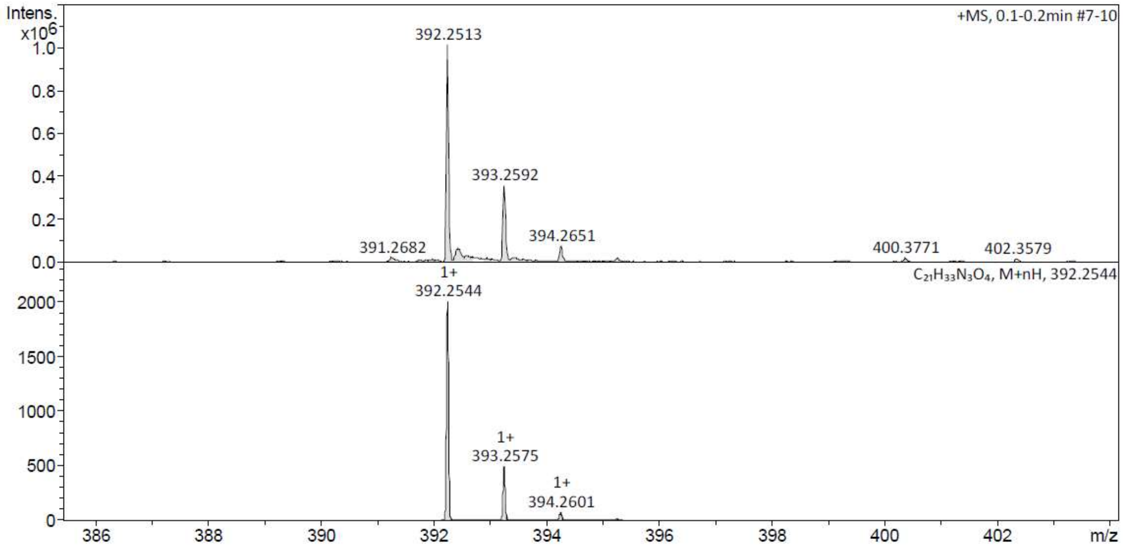

Figure S6

HRMS-MS ESI$^+$ Spectrum of M4

Display Report

Analysis Info

Analysis Name	D:\Data\Jose Correa Basurto\112218_M-4_392msms_.d	Acquisition Date	11/22/2018 3:08:37 PM
Method	Tune low 25-sep-2018.m	Operator	Daniel Arrieta
Sample Name	112218_M-4_392msms_	Instrument	micrOTOF-Q 228888.10392
Comment			

Acquisition Parameter

Source Type	ESI	Ion Polarity	Positive	Set Nebulizer	0.4 Bar
Focus	Active	Set Capillary	4500 V	Set Dry Heater	180 °C
Scan Begin	50 m/z	Set End Plate Offset	-500 V	Set Dry Gas	4.0 l/min
Scan End	3000 m/z	Set Collision Cell RF	150.0 Vpp	Set Divert Valve	Source

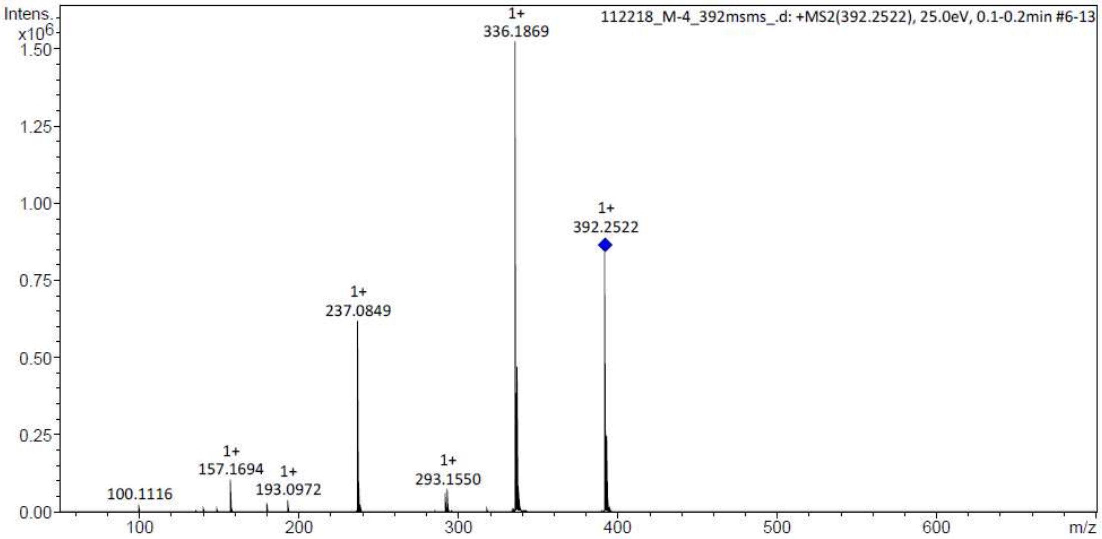

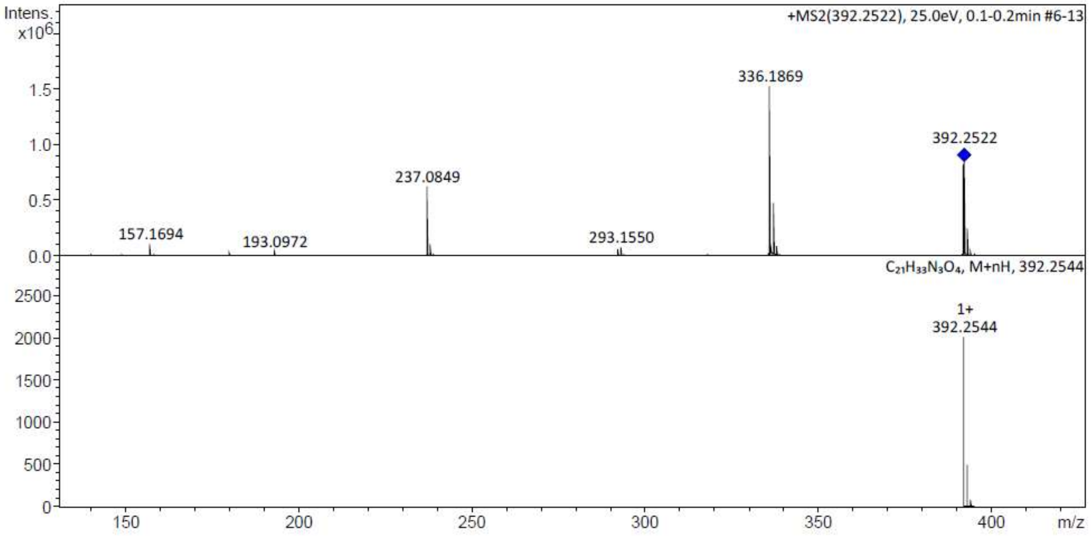

Figure S7

FTIR Spectrum of M4

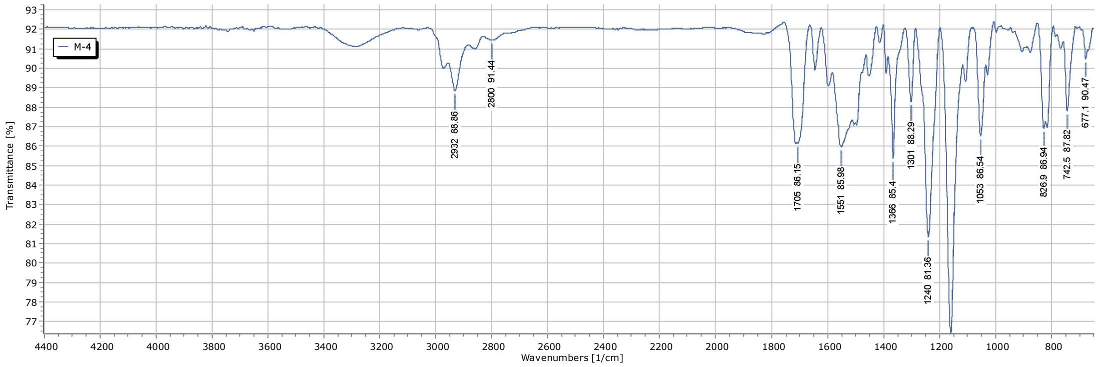

Supplement: Supplementary file 1 [file molecules-25-05009-s001.pdf]
